# Correlation between normally aerated lung and respiratory system compliance at clinical high positive end-expiratory pressure in patients with COVID-19

**DOI:** 10.1038/s41598-024-64622-3

**Published:** 2024-06-24

**Authors:** Keishi Ogura, Ryuichi Nakayama, Naofumi Bunya, Shinshu Katayama, Naoya Yama, Yuya Goto, Keigo Sawamoto, Shuji Uemura, Eichi Narimatsu

**Affiliations:** 1 Division of Radiology and Nuclear Medicine, Sapporo Medical University Hospital, Sapporo, Japan; 2 Department of Emergency Medicine, Sapporo Medical University School of Medicine, 291, Minami 1-jo Nishi 16-chome, Chuo-ku, Sapporo, 060-8556 Japan; 3 Division of Intensive Care, Department of Anesthesiology and Intensive Care Medicine, Jichi Medical University School of Medicine, Shimotsuke, Tochigi 329-0498 Japan; 4 Department of Diagnostic Radiology, Sapporo Medical University School of Medicine, Sapporo, Japan; 5 Department of Intensive Care Medicine, School of Medicine, Sapporo Medical University, Sapporo, Hokkaido Japan

**Keywords:** Respiratory distress syndrome, Mechanical ventilation, Coronavirus disease 2019, Respiratory system compliance, Computed tomography, Recruitability, Respiratory distress syndrome, Computed tomography

## Abstract

Normally aerated lung tissue on computed tomography (CT) is correlated with static respiratory system compliance (C_rs_) at zero end-expiratory pressure. In clinical practice, however, patients with acute respiratory failure are often managed using elevated PEEP levels. No study has validated the relationship between lung volume and tissue and C_rs_ at the applied positive end-expiratory pressure (PEEP). Therefore, this study aimed to demonstrate the relationship between lung volume and tissue on CT and C_rs_ during the application of PEEP for the clinical management of patients with acute respiratory distress syndrome due to COVID-19. Additionally, as a secondary outcome, the study aimed to evaluate the relationship between CT characteristics and C_rs_, considering recruitability using the recruitment-to-inflation ratio (R/I ratio). We analyzed the CT and respiratory mechanics data of 30 patients with COVID-19 who were mechanically ventilated. The CT images were acquired during mechanical ventilation at PEEP level of 15 cmH_2_O and were quantitatively analyzed using Synapse Vincent system version 6.4 (Fujifilm Corporation, Tokyo, Japan). Recruitability was stratified into two groups, high and low recruitability, based on the median R/I ratio of our study population. Thirty patients were included in the analysis with the median R/I ratio of 0.71. A significant correlation was observed between C_rs_ at the applied PEEP (median 15 [interquartile range (IQR) 12.2, 15.8]) and the normally aerated lung volume (r = 0.70 [95% CI 0.46–0.85], *P* < 0.001) and tissue (r = 0.70 [95% CI 0.46–0.85], *P* < 0.001). Multivariable linear regression revealed that recruitability (Coefficient = − 390.9 [95% CI − 725.0 to − 56.8], *P* = 0.024) and C_rs_ (Coefficient = 48.9 [95% CI 32.6–65.2], *P* < 0.001) were significantly associated with normally aerated lung volume (R-squared: 0.58). In this study, C_rs_ at the applied PEEP was significantly correlated with normally aerated lung volume and tissue on CT. Moreover, recruitability indicated by the R/I ratio and C_rs_ were significantly associated with the normally aerated lung volume. This research underscores the significance of C_rs_ at the applied PEEP as a bedside-measurable parameter and sheds new light on the link between recruitability and normally aerated lung.

Computed tomography (CT) of patients with acute respiratory distress syndrome (ARDS) demonstrates a “baby lung” condition with areas of reduced aeration, showing preferential distribution of densities to dependent lung areas and relative sparing of the non-dependent areas^1^.

Gattinoni et al. reported that normally aerated lung tissue on CT correlated with static respiratory system compliance (C_rs_) of the pressure–volume curve (PV curve) with low-flow inflation from zero end-expiratory pressure (ZEEP)^2^. In clinical practice, however, patients with acute respiratory failure are often managed using elevated positive end-expiratory pressure (PEEP) levels^3^. One method of PEEP titration is the "best compliance" approach, which assesses C_rs_ under applied PEEP during a decremental PEEP trial. The underlying principle posits that an increase in C_rs_ with lower PEEP suggests a reduction in the number of hyper-inflated alveoli, whereas a decrease in C_rs_ with lower PEEP indicates a rise in the number of collapsed alveoli^4,5^. To the best of our knowledge, no study has validated the relationship between lung volume and tissue on CT and C_rs_ at the applied PEEP.

Lung aeration and inflation vary depending on recruitability, which reflects the reactivity via high airway pressure or PEEP^6,7^. Recruitability can be assessed at the bedside by means of the recruitment-to-inflation ratio (R/I ratio) using respiratory mechanics^8^. The relationship between lung CT and C_rs_ in the context of recruitability remains unexplored.

We hypothesized that C_rs_ would also be correlated with aerated lung volume and tissue on CT at the applied PEEP but that the situation would vary depending on recruitability. This study aimed to demonstrate the relationship between lung on CT and C_rs_ during the application of PEEP in clinical practice for the management of ARDS patients with COVID-19. Additionally, as a secondary outcome, the study aimed to evaluate the relationship between CT characteristics and C_rs_, considering the R/I ratio.

## Methods

### Study design

This was a secondary analysis of the data obtained from a single-center cohort study of patients with COVID-19 who underwent invasive mechanical ventilation in the intensive care unit (ICU) of the Department of Emergency Medicine, Sapporo Medical University, Sapporo, Hokkaido, Japan, between January 1, 2021, and September 30, 2021^9^. This study was conducted in accordance with the tenets of the Declaration of Helsinki and was approved by the Ethics Committee of our institution (Approval Code: 342-1130) on December 19, 2022. Owing to the retrospective nature of the study, the requirement for obtaining informed consent was waived. The patients and their kin were provided with the option to withdraw consent at their discretion.

### Patient population

The inclusion criteria for this study were as follows: (1) age ≥ 18 years, (2) ventilated patients with ARDS due to COVID-19, (3) patients with R/I ratio measurements, and (4) patients with plain CT acquired in apneic state at a PEEP level of 15 cmH_2_O within 24 h before or after R/I ratio measurements.

### PEEP setting

The initial PEEP setting strategy for patients with acute respiratory failure at our institution is to use a higher PEEP/F_i_O_2_ strategy^3^ or set PEEP at 15 or 18 cmH_2_O in preparation for R/I ratio measurements^8^, provided the patient remains hemodynamically stable.

### C_rs_, R/I ratio, and airway opening pressure

C_rs_ was calculated by dividing the tidal volume by driving pressure at the clinically applied PEEP^10^, not using low-flow inflation PV curve.

The R/I ratio was derived by reducing PEEP from a higher to a lower pressure (from 15 to 5 cmH_2_O, or 18–8 cmH_2_O) using a single breath method after confirming the presence of an airway opening pressure (AOP) of > 5 cmH_2_O. In the case without AOP, for example, the measured change in end-expiratory lung volume between two PEEP levels (measured ΔEELV) is determined from the exhaled breath when the PEEP is dropped from 15 to 5 cmH_2_O. Predicted ΔEELV is calculated by multiplying the C_rs_ at low PEEP by the pressure over which recruitment is assessed (ΔPrec). Recruited volume (ΔVrec) is calculated by subtracting predicted ΔEELV from measured ΔEELV. Compliance of the recruited lung (Crec) is defined as the ΔVrec divided by the ΔPrec. The R/I ratio is defined as the Crec divided by the C_rs_ at low PEEP (Supplementary Fig. 1).

AOP was identified as the lower inflection point of the quasi-static PV curve with compliance as low as 1.5–2.5 mL/cmH_2_O above 5 cmH_2_O using a ventilator automatic application (P/V tool; Hamilton Medical AG, Bonaduz, Switzerland) for low-flow inflation and deflation with a constant pressure variation of 2 cmH_2_O/s^9,11^. All evaluations were performed in the supine flat position under passive ventilation with sedation and neuromuscular blockade. If the PEEP is set to 5 cmH_2_O or ZEEP for a patient with ARDS who has a higher AOP than 5 cmH_2_O, pressure is required to open the distal airway ("wasting" driving pressure), which may lead to misinterpretation of C_rs_^12^. In the patients of this study with AOP higher than 5 cmH_2_O, C_rs_ was calculated with the clinical PEEP set to exceed the AOP, thereby eliminating this concern.

### CT scan evaluation

CT of the chest was performed using an 80-row multi-slice CT scanner (Aquilion Prime; Canon Medical Systems, Otawara, Tochigi, Japan). The lungs were imaged from the apex to the diaphragm during expiratory breath-holding on a mechanical ventilator at a PEEP level of 15 cmH_2_O. PEEP 15 cmH_2_O was applied for at least 30 min prior to imaging to minimize the risk of derecruitment.

The CT images were quantitatively analyzed using the Synapse Vincent system version 6.4 (Fujifilm Corporation, Tokyo, Japan). Slices of 1-mm thickness were outlined using system-assisted and manual methods after excluding the mediastinum, hilar vessels, and trachea. The voxels in the whole lungs, which had a CT number (Hounsfield unit scale [HU]), were classified into four groups according to the CT number: nonaerated (+ 100 HU to − 100 HU), poorly aerated (− 101 to − 500 HU), normally aerated (− 501 to − 900 HU), and hyperinflated (− 1000 HU to − 901 HU) (Supplementary Fig. 2)^2^. Assuming that the specific lung weight was equal to 1, the lung tissue weight was calculated using voxel CT number and voxel volume. The formula applied was:

$${\text{Tissue}}\;{\text{weight}} = \left( {{1}{-}{\text{CT}}\;{\text{number/}} - {1}000} \right)*{\text{Voxel}}\;{\text{volume}}$$

$${\text{Lung}}\;{\text{volume}} = ({\text{CT}}\;{\text{number}}/ - {1}000)*{\text{Voxel}}\;{\text{volume}}$$

Based on previous studies, we defined residual inflated lung tissue as follows^2,13,14^:

$${\text{Expected}}\;{\text{normal}}\;{\text{lung}}\;{\text{tissue}}\left( {\text{g}} \right) = - 1806.1 + 1633.7 \times {\text{Height}}\left( {\text{m}} \right)$$

$${\text{Residual}}\;{\text{inflated}}\;{\text{lung}}\;{\text{tissue}}\left( \% \right) = {\text{Normally}}\;{\text{aerated}}\;{\text{tissue}}/{\text{``Expected''}}\;{\text{normal}}\;{\text{lung}}\;{\text{tissue}}$$

### Data collection and measurements

The following baseline patient characteristics were collected: “age, sex, height, weight, body mass index (BMI), and preexisting medical conditions. The following parameters were obtained 24 h before or after the CT examination: the PaO_2_/F_i_O_2_ ratio (P/F ratio), duration of ventilation, Sequential Organ Failure Assessment (SOFA) scores, tidal volume divided by the predicted body weight, PEEP, P_plat_, C_rs_, and R/I ratio. Respiratory data measurements were conducted just prior to the R/I ratio assessment to avoid the risk of derecruitment.

### Data analysis

The primary outcome assessed in this study was the correlation between C_rs_ at the applied PEEP and the lung volume (normally aerated, poorly aerated, and hyperinflated lung volume) and tissue on CT (normally aerated, poorly aerated, and nonaerated lung tissue). The secondary outcome was to validate the association between C_rs_ and normally aerated lung volume and tissue, incorporating the analysis of recruitability using linear regression. Recruitability was stratified into two groups, high and low recruitability, based on the median R/I ratio of our study population, following the methodology of Chen et al.^8^. Moreover, we added post-hoc analyses that categorized recruitability based on the median value of the ΔVrec.

### Statistical analysis

Data are expressed as the median and interquartile range (IQR). The correlation between the variables was assessed using Pearson’s correlation coefficient with 95% confidence intervals (CIs). The analyses were two-sided, and a *P*-value < 0.05 were considered statistically significant. The type I errors for the primary and secondary outcomes were controlled using the Bonferroni method. For the primary outcome, a *P*-value of less than 0.0083 was considered statistically significant, while for the secondary outcome, a *P*-value of less than 0.025 was considered statistically significant. There was no prespecified approach for multiple comparison except for the primary outcome and secondary outcome. Therefore, reported point estimates for correlation matrix were not adjusted and thus should be interpreted with caution. All analyses were performed using R software version 4.2.2. (The R Foundation for Statistical Computing, Vienna, Austria).

### Ethics approval and consent to participate

This study was approved by the Ethics Committee of Sapporo Medical University (342-1130) on December 19, 2022. The requirement for informed consent was waived due to the retrospective design of the study.

## Results

### Enrolment and baseline characteristics

**Figure 1:**
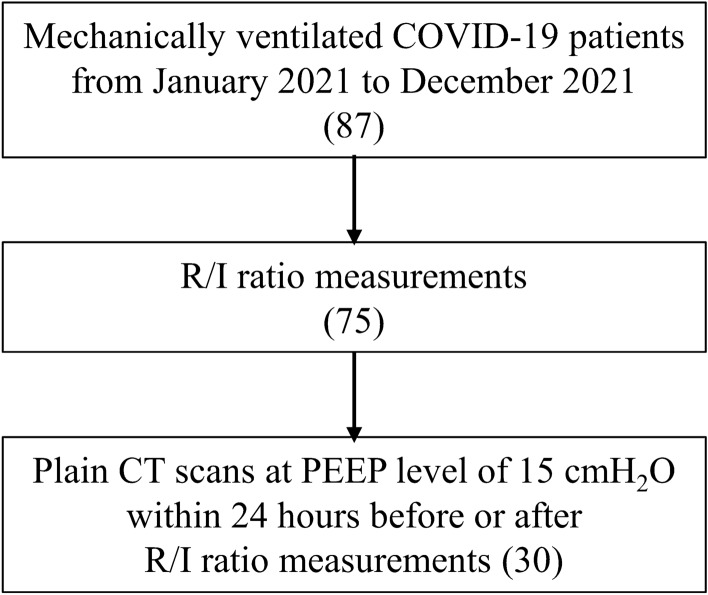
Flow diagram of the study patients. COVID-19, coronavirus disease 2019; R/I, recruitment-to-inflation; CT, computed tomography; PEEP, positive end-expiratory pressure.

Thirty patients were included in the analysis (Fig. 1). Table 1 presents the baseline patient characteristics and the division of the groups according to the median R/I ratio (0.71). Four patients (13.3%) had airway closure phenomenon: two patients each with an AOP of 6 cmH_2_O and 8 cmH_2_O. The clinical PEEP was higher than the AOP in all four cases.

**Table 1:** Patient characteristics and the division of the groups according to the median R/I ratio (0.71).

	Total	R/I ratio > 0.71	R/I ratio ≤ 0.71
	N = 30	N = 15	N = 15
Age, years	63.5 [51.0, 66.0]	58.0 [48.5, 65.0]	65.0 [56.5, 69.0]
Sex, men	22 (73.3)	12 (80.0)	10 (66.7)
Height, cm	169.0 [162.0, 172.8]	170.0 [166.2, 172.5]	164.0 [159.0, 173.0]
Body weight, kg	77.3 [67.0, 89.7]	84.0 [71.5, 89.4]	71.0 [63.5, 89.3]
Body mass index, kg/m^2^	27.9 [23.8, 29.6]	28.4 [25.9, 29.9]	26.2 [22.7, 29.4]
Predicted body weight, kg	65.1 [55.3, 68.5]	66.0 [61.9, 68.3]	60.6 [53.8, 68.7]
KL-6 at admission, U/mL	464.0 [355.5, 745.5]	415.0 [359.0, 609.0]	565.0 [388.5, 953.0]
Receiving ECMO, n (%)	4 (13.3)	2 (13.3)	2 (13.3)
Receiving prone position, n (%)	29 (96.7)	15 (100.0)	14 (93.3)
Receiving high-flow nasal cannula before mechanical ventilation, n (%)	3 (10.0)	0 (0.0)	3 (20.0)
Survival at discharge, n (%)	28 (93.3)	15 (100.0)	13 (86.7)
Pre-existing conditions, n (%)			
COPD	15 (50.0)	5 (33.3)	10 (66.7)
Hypertension	12 (40.0)	5 (33.3)	7 (46.7)
Diabetes	26 (86.7)	13 (86.7)	13 (86.7)
SOFA score at R/I ratio measurement	5.5 [3.2, 7.0]	5.0 [3.5, 6.5]	7.0 [3.5, 8.0]
PaO_2_/F_i_O_2_ at recruitability assessment, mmHg	119.7 [102.2, 193.8]	165.0 [106.5, 234.0]	108.0 [91.6, 153.8]
Respiratory data at R/I ratio measurement			
TV, mL	395.0 [350.0, 416.8]	400.0 [367.5, 454.0]	370.0 [328.5, 400.0]
TV/PBW, mL/kg	6.2 [5.9, 6.8]	6.1 [6.0, 7.0]	6.2 [5.7, 6.6]
Respiratory rate, b/min	16.0 [15.0, 18.0]	16.0 [14.5, 18.0]	15.0 [15.0, 18.0]
P_plat_, cmH_2_O	25.0 [23.0, 27.8]	25.0 [23.5, 27.0]	25.0 [22.0, 27.5]
PEEP, cmH_2_O	15.0 [12.2, 15.8]	15.0 [15.0, 16.0]	13.0 [12.0, 15.0]
C_rs_, mL/cmH_2_O	38.3 [28.0, 44.3]	43.3 [34.2, 49.4]	35.5 [26.3, 40.5]
AOP > 5 cmH_2_O, n (%)	4 (13.3)	2 (13.3)	2 (13.3)
R/I ratio	0.71 [0.52, 0.88]	0.88 [0.82, 1.35]	0.52 [0.42, 0.62]
∆Vrec, mL	283.1 [191.0, 348.5]	356.0 [314.0, 493.2]	189.0 [129.7, 223.6]
Measured ∆EELV, mL	634.0 [518.0, 761.8]	726.0 [625.5, 934.0]	556.0 [425.5, 643.5]
Crec, mL/cmH_2_O	28.3 [20.1, 34.9]	35.6 [31.4, 55.2]	21.1 [13.3, 22.9]
Lung analyses data on CT			
Interval between onset and CT scans, day	10.0 [7.2, 12.0]	10.0 [7.0, 12.0]	10.0 [8.0, 13.0]
Hyperinflated lung tissue, g	11.3 [5.7, 22.0]	12.8 [8.8, 23.5]	7.3 [2.8, 20.3]
Normally aerated lung tissue, g	515.7 [380.1, 673.6]	515.5 [395.1, 624.6]	562.4 [337.6, 672.2]
Poorly aerated lung tissue, g	488.0 [342.5, 553.1]	472.3 [380.3, 595.7]	503.7 [348.5, 543.5]
Nonaerated lung tissue, g	221.9 [116.1, 375.5]	184.8 [134.2, 361.8]	250.4 [120.1, 384.5]
Hyperinflated lung volume, mL	147.8 [70.6, 294.4]	99.6 [31.1, 241.6]	187.7 [121.2, 314.3]
Normally aerated lung volume, mL	1422.9 [903.9, 1792.7]	1375.1 [814.1, 1859.0]	1470.7 [1008.0, 1742.2]
Poorly aerated lung volume, mL	246.0 [164.5, 267.5]	241.1 [155.1, 262.1]	258.3 [193.6, 285.1]
Nonaerated lung volume, mL	6.2 [4.3, 8.7]	6.3 [4.5, 8.4]	5.4 [4.2, 9.2]
Residual inflated lung tissue, %	56.5 [39.6, 69.6]	58.9 [36.8, 67.9]	54.0 [42.1, 72.5]

Continuous variables were expressed as median [interquartile range]. Categorical variables were expressed as numbers and proportions.

KL-6, Krebs von den Lungen-6; ECMO, extracorporeal membrane oxygenation; COPD, chronic obstructive pulmonary disease; SOFA, Sequential Organ Failure Assessment; TV, tidal volume; PBW, predicted body weight; P_plat_, plateau pressure; PEEP, positive end-expiratory pressure; C_rs_, respiratory system compliance; AOP, airway opening pressure; R/I ratio, recruitment-to-inflation ratio; ΔVrec, recruited volume; ∆EELV, change in end-expiratory lung volume; Crec, compliance of the recruited lung; CT, computed tomography.

### ***C***_***rs***_ and CT

**Figure 2:**
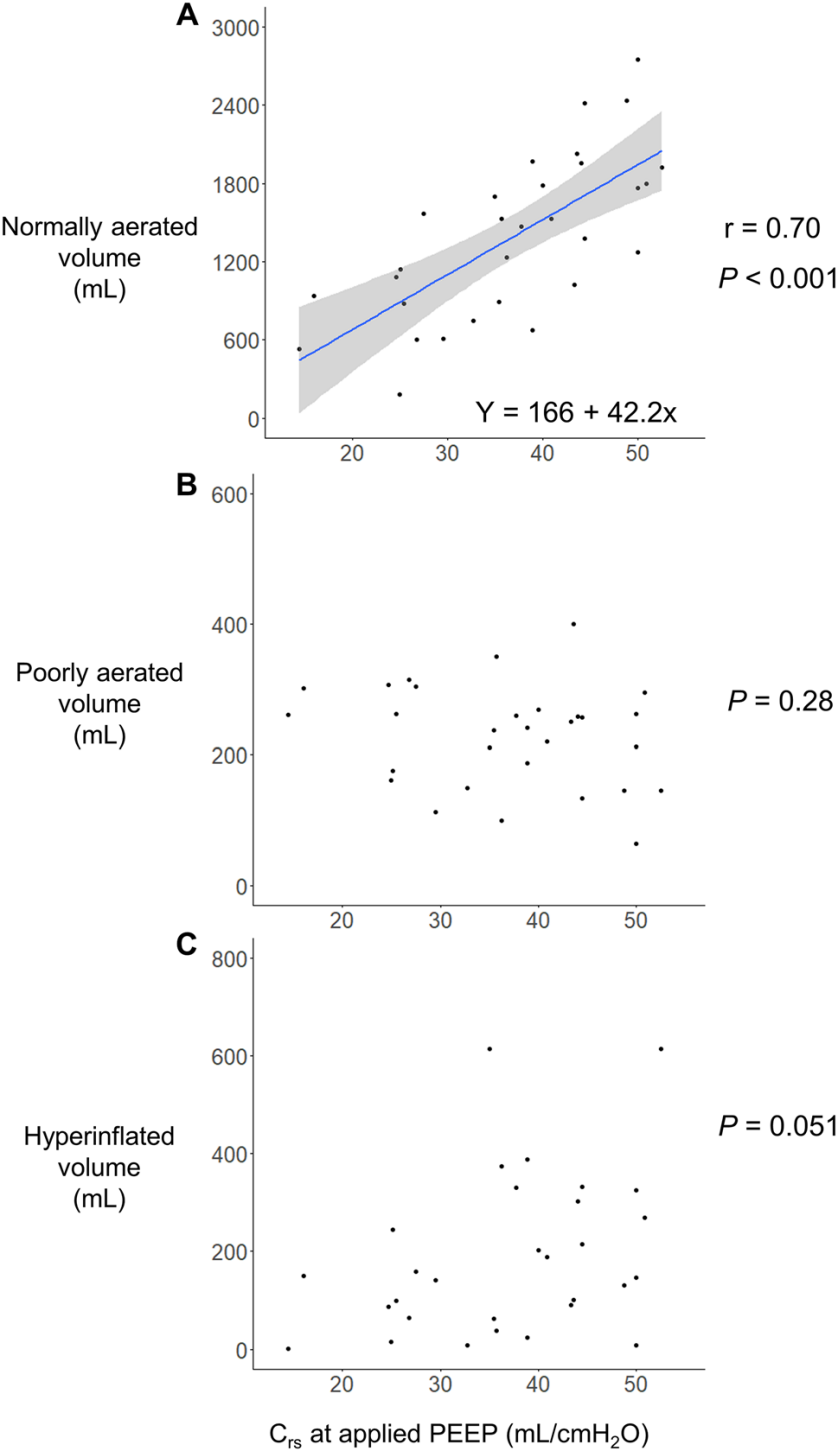
Scatter diagrams of the relationship between the C_rs_ at the clinical setting of PEEP and lung volume on CT. C_rs_ at the clinical setting of PEEP was significantly correlated with normally aerated lung volume (r = 0.70, *P* < 0.001, Fig. 2A). Linear regression analysis indicated that for every unit increase in C_rs_, the normally aerated lung volume increased by 42.2 mL, with a y-intercept of 166.0 m. C_rs_ at the clinical setting of PEEP did not correlate with the poorly aerated and hyperinflated air volume (Fig. 2B and C). C_rs_, static respiratory system compliance; CT, computed tomography; PEEP, positive end-expiratory pressure.

**Figure 3:**
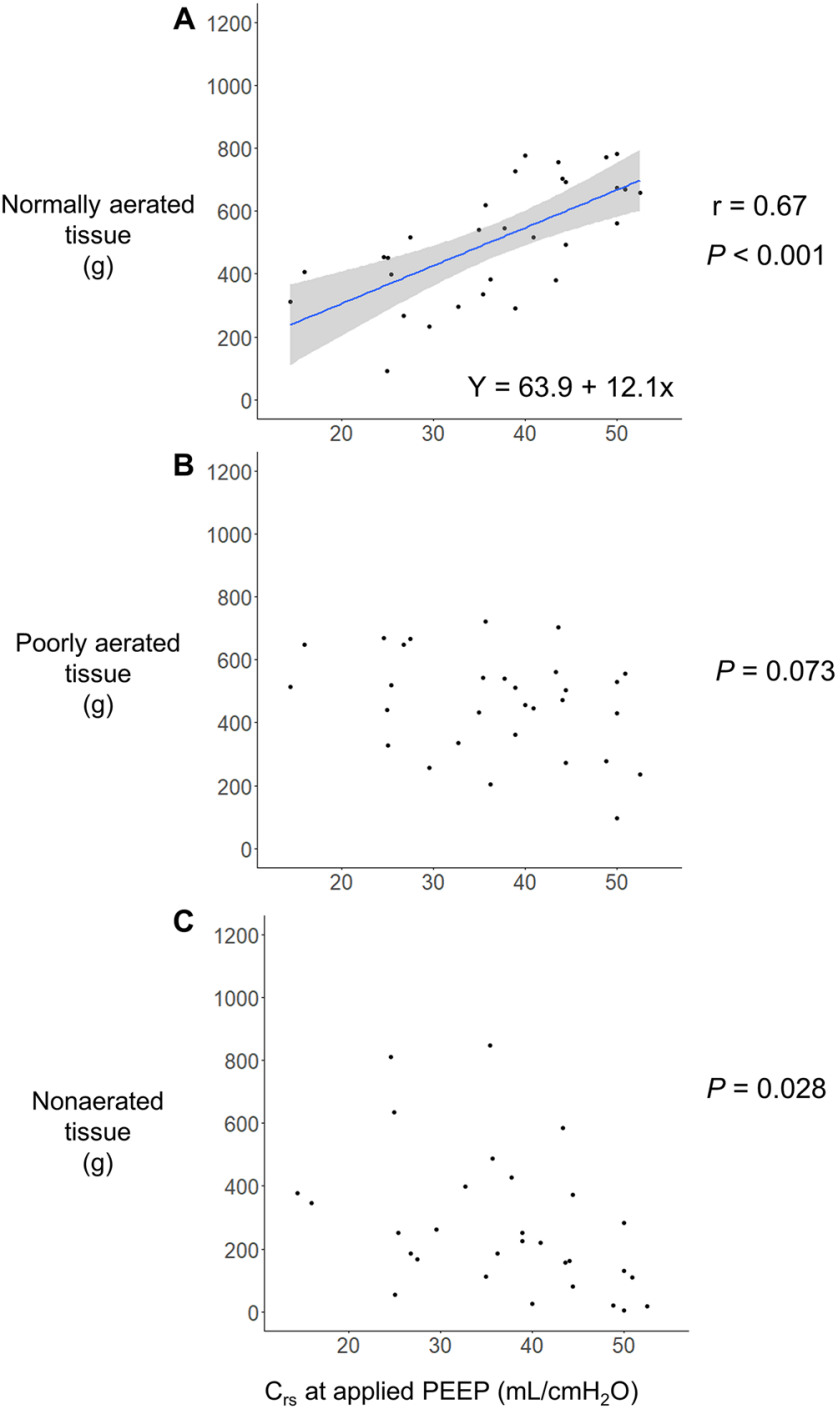
Scatter diagrams of the relationship between the C_rs_ at the clinical setting of PEEP and lung tissue on CT. C_rs_ at the clinical setting of PEEP was significantly correlated with normally aerated tissue (r = 0.67, *P* < 0.001, Fig. 3A). Linear regression analysis indicated that for every unit increase in C_rs_, the normally aerated tissue increased by 12.1 g, with a y-intercept of 63.9 g. C_rs_ at the clinical setting of PEEP did not correlate with the poorly aerated and nonaerated tissue (Fig. 3B,C). C_rs_, static respiratory system compliance; CT, computed tomography; PEEP, positive end-expiratory pressure.

A significant correlation was observed between C_rs_ at the applied PEEP (median 15.0 [IQR 12.2, 15.8]) and the normally aerated lung volume (r = 0.70 [95% CI 0.46–0.85], *P* < 0.001, Fig. 2A) and tissue (r = 0.67 [95% CI 0.41–0.83], *P* < 0.001, Fig. 3A). Linear regression analysis indicated that for every unit increase in C_rs_, the normally aerated lung volume increased by 42.2 mL, with a y-intercept of 166.0 mL, and the normally aerated tissue increased by 12.1 g, with a y-intercept of 63.9 g. However, no correlation was observed between C_rs_ and the poorly aerated and hyperinflated airvolume (Fig. 2B,C) or the poorly aerated and nonaerated tissue (Fig. 3B,C).

Multivariable linear regression revealed that recruitability (Coefficient = − 390.9 [95% CI − 725.0 to − 56.8], *P* = 0.024) and C_rs_ (Coefficient = 48.9 [95% CI 32.6–65.2], *P* < 0.001) were significantly associated with normally aerated lung volume (R-squared: 0.58). On the other hand, recruitability was not significantly associated with normally aerated tissue (Coefficient = − 107.8 [95% CI [− 214.8 to − 0.7], *P* = 0.048, R-squared: 0.49) (Table 2).

**Table 2:** Multivariable linear regression analysis validating the association between respiratory system compliance and normally aerated lung volume and tissue, incorporating recruitability adjusted based on the median R/I ratio of the study population.

	Coefficient [95% CI]	
	Normally aerated volume (mL)	Normally aerated tissue (g)
Intercept	− 216.8 [− 803.5, 369.9]	49.9 [− 138.1, 237.9]
Recruitability (R/I ratio > 0.71)	− 390.9* [− 725.0, − 56.8]	− 107.8 [− 214.8, − 0.7]
Respiratory system compliance	48.9*** [32.6, 65.2]	13.9*** [8.7, 19.1]

The estimates of the regression coefficients are showed with 95% confidence interval by multivariate linear regression analysis. Recruitability was stratified into two groups, high and low recruitability, based on the median recruitment-to-inflation ratio (R/I ratio) (0.71) of our study population.

CI, confidence interval; R/I ratio, recruitment-to-inflation ratio.

*P < 0.025 **P < 0.005 ***P < 0.0005.

**Figure 4:**
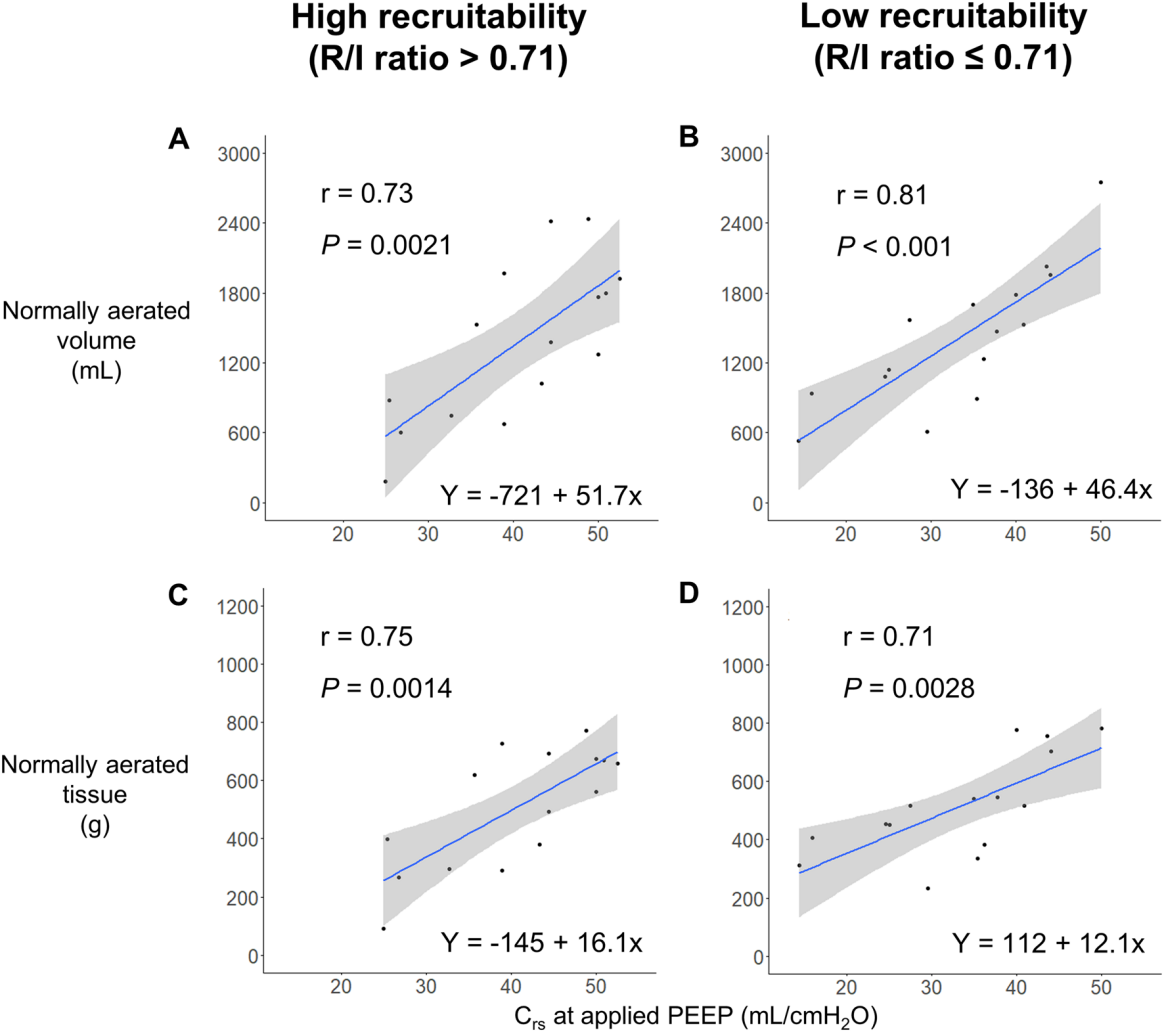
Correlation of the C_rs_ at the clinical setting of PEEP with normally aerated lung volume and tissue when divided into two groups according to recruitability indicated by the median R/I ratio of 0.71. The relationship between C_rs_ at the clinical setting of PEEP and the normally aerated lung volume and tissue was more strongly correlated when divided by the median R/I ratio of 0.71. For lung volume, the high recruitability group had a correlation coefficient (r) of 0.73 (*P* = 0.0021), and the low recruitability group had an r of 0.81 (*P* < 0.001) (Fig. 4**A**,**B**). For tissue, the high recruitability group had an r of 0.75 (*P* = 0.0014), and the low recruitability group had an r of 0.71 (*P* = 0.0028) (Fig. 4**C**,**D**). C_rs_, static respiratory system compliance; PEEP, positive end-expiratory pressure; R/I, recruitment-to-inflation ratio.

When stratified by the median R/I ratio of 0.71, a stronger correlation was observed between C_rs_ at the applied PEEP and the normally aerated lung volume (High recruitability group: r = 0.73 [95% CI 0.34–0.90], *P* = 0.0021, Low recruitability group: r = 0.81 [95% CI 0.51–0.94], *P* < 0.001, Fig. 4A,B), and the normally aerated tissue (High recruitability group: r = 0.75 [95% CI 0.38–0.91], *P* = 0.0014, Low recruitability group: r = 0.71 [95% CI 0.31–0.90], *P* = 0.0028, Fig. 4C,D). No relationship was observed between C_rs_ and the poorly aerated and hyperinflated volume, or the poorly aerated and nonaerated tissue.

**Figure 5:**
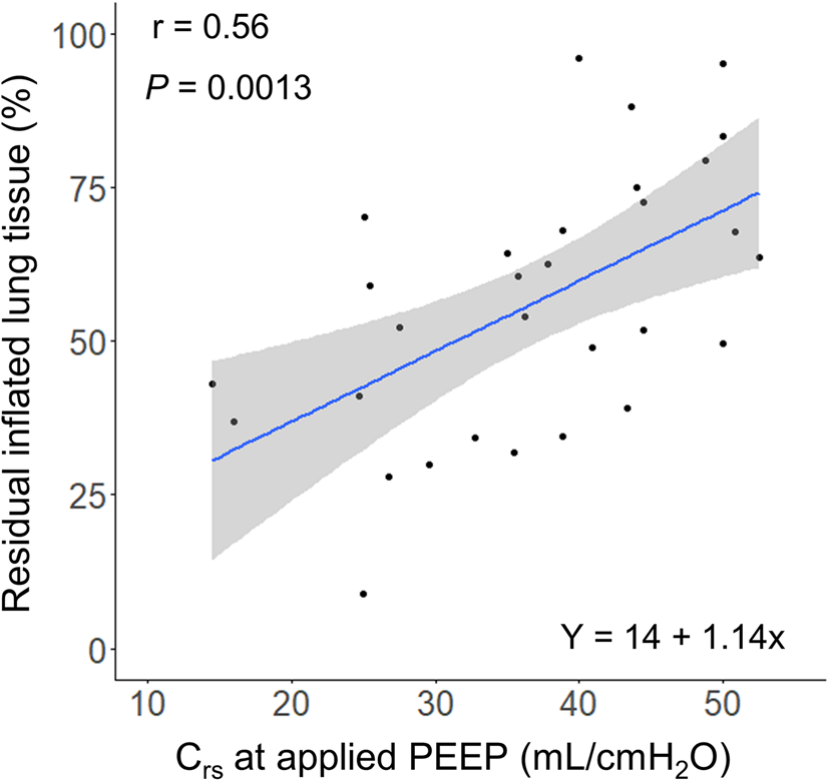
Correlation between the C_rs_ at the applied PEEP and residual inflated lung tissue (r = 0.56, *P* = 0.0013). C_rs_, static respiratory system compliance; PEEP, positive end-expiratory pressure.

A moderate correlation was observed between C_rs_ at the clinical setting of PEEP and the residual inflated lung tissue (r = 0.56 [95% CI 0.25–0.77], *P* = 0.0013, Fig. 5) and the correlation was found to be stronger when grouped by the median R/I ratio (High recruitability group: r = 0.65 [95% CI 0.21–0.87], *P* = 0.0083, Low recruitability group: r = 0.63 [95% CI 0.18–0.86], *P* = 0.011, Supplementary Fig. 3A and B).

Supplementary Table 3 presents the correlation matrix, including the respiratory mechanics and the lung analysis items on CT. No correlation was observed between any of the lung analysis items on CT and the R/I ratio. A moderately positive correlation was observed between the P/F ratio and the normally aerated lung volume (r = 0.47 [95% CI 0.13–0.71], *P* = 0.010). A significantly negative correlation was observed between the nonaerated and normally aerated tissue and between the nonaerated and residual inflated lung tissue (respectively, r = − 0.57 [95% CI − 0.77 to − 0.26], *P* = 0.0011 and r = − 0.66 [95% CI − 0.82 to − 0.39], *P* < 0.001).

### Post-hoc analyses

Multivariable linear regression revealed that recruitability divided by the median ΔVrec was not associated with normally aerated lung volume (Coefficient = − 4.3 [95% CI [− 423.3 to 368.9], *P* = 0.89, R-squared: 0.46) and tissue (Coefficient = − 27.2 [95% CI [− 128.4 to 119.7], *P* = 0.94, R-squared: 0.41) (Supplementary Table 2).

When stratified by the median ΔVrec, only in the high ΔVrec group was there a significant correlation between normally aerated lung and C_rs_ (Supplementary Fig. 4).

## Discussion

### Key findings

This study revealed that C_rs_ at the applied PEEP was significantly correlated with normally aerated lung volume and tissue on CT. Moreover, recruitability was statistically significantly associated with the normally aerated lung air volume. Owing to the substantial resources required and risks associated with transporting patients on mechanical ventilation, CT evaluation is not a feasible procedure that can be performed routinely for patients with ARDS^15^. Therefore, this study not only reaffirms the importance of C_rs_ at the applied PEEP as a parameter that can be determined at the bedside but also newly elucidates the relationship between recruitability and normally aerated lung. This study also provided clinical validity to the relationship between C_rs_ and the “baby lung” at PEEP settings based on the “best compliance” method.

### Relationship with previous studies

A previous study reported that starting compliance, which is the ratio between the first 100 mL of inflation from ZEEP and the corresponding pressure, correlated with the normally aerated tissue and residual inflated lung at 5 cmH_2_O PEEP. A higher PEEP is commonly used for patients with moderate or severe ARDS^3,16^. C_rs_ at a higher level of PEEP has not been validated using lung analyses on CT. In the present study, Pearson's correlation coefficient for C_rs_ at the clinical setting of PEEP and the normally aerated tissue was 0.67 (Fig. 3A). This value was lower than the correlation of 0.83 reported for starting compliance and the normally aerated tissue by Gattinoni et al. In contrast, it was close to the correlation of 0.64 reported for inflation compliance (defined as the maximum slope of the static PV curve) and the normally aerated tissue at 15 cmH_2_O PEEP in the same study. Inflation compliance is the maximum slope of the PV curve, the lowest pressure of which is expressed as the "best" PEEP (mean 11.1 cmH_2_O). Inflation compliance appears to be the C_rs_ from this pressure. Since the clinical setting of PEEP in this study (mean 14.2 cmH_2_O) was closer to this "best" PEEP than to ZEEP, the results of this study may be closer to the results of the previous study on inflation compliance than those of the study on starting compliance^2^.

As illustrated in Fig. 5, a correlation was observed between C_rs_ at the applied PEEP and the residual inflated lung tissue (r = 0.56), representing the relative size of the "baby lung" to the normal lung (“expected” normal lung tissue). However, the correlation coefficient was lower than that previously reported (r = 0.86)^2^. This discrepancy could be attributed to the calculation of the "expected" normal lung tissue based on a Spanish study^17^, which measured the functional residual capacity in normal participants, potentially reflecting racial differences. Furthermore, a stronger correlation was observed when the patients were grouped according to the recruitability (Supplementary Fig. 3). The patients in the previous study may have exhibited homogeneous recruitability.

### Relationship with recruitability

In this study, recruitability defined by R/I ratio and C_rs_ were significantly associated with the normally aerated lung volume (Table 2) and the correlation between C_rs_ at the applied PEEP and the normally aerated volume and tissue on CT was stronger when the patients were stratified into two groups according to recruitability indicated by the median R/I ratio (Fig. 4). In contrast, the R/I ratio did not correlate with the lung analysis of CT at PEEP 15 cmH_2_O or with the respiratory parameters of the P/F ratio or C_rs_ (Supplementary Table 3). This finding suggests that the R/I ratio, as well as P/F ratio and C_rs_, is a different lung parameter from lung analysis at the same PEEP on CT. Recruitability defined by respiratory mechanics like R/I ratio refers to the ability to improve lung inflation via high airway pressure or PEEP^7^. In other words, variations in recrutability could alter the degree of lung aeration that a PEEP of 15 cmH_2_O produces in a patient. This study suggests that the lung status on CT was different for each recruitability level.

The stronger correlation between C_rs_ and normally aerated lung volume and tissue, when patients were divided into two groups according to the R/I ratio, suggests different phenotypes of respiratory failure due to COVID-19 influenced by recruitability. In other words, the baby lung status may vary depending on whether the patient has Type H with high recruitability or Type L with low recruitability^18^. Even if C_rs_ remains the same, a patient with high recruitability results in a lower intercept, as shown in Fig. 4, indicating that the baby lung may be smaller. However, the CI was wider due to the small sample size, necessitating a larger prospective study to confirm these findings.

The lack of association between normalized aerated lung and the two groups divided by the median ΔVrec (Supplementary Table 2 and Supplementary Fig. 4) may be due to the difficulty in classifying phenotypes based solely on ΔVrec. A previous study reported that ΔVrec is greater in healthy individuals than in patients with ARDS^19^. On the other hand, for the R/I ratio, standardization by C_rs_ at low PEEP can identify patients with high C_rs_ and large ΔVrec as Type L, and those with low C_rs_ and large ΔVrec as Type H. Post-hoc analyses indicated that it is difficult to determine the type of recruitability based solely on whether ΔVrec is high or low.

### Limitations

This study has several limitations. First, this was a secondary analysis of a single-center study with a small sample size in patients with respiratory failure due solely to COVID-19. A prospective observational study will be required to further build on these results. Second, in this study, we did not obtain data separating respiratory compliance into chest wall and lung components, and it is possible that the chest wall component may have modified the results. Third, we lacked CT data for PEEP at 5 cmH_2_O owing to infection control and resource issues. Lung analysis at a PEEP of 5 cmH_2_O may have enabled comparison with the present results. Fourth, the analysis included patients with a window period of up to 24 h, incorporating those who underwent CT scans within this time frame after the R/I ratio and C_rs_ measurements. This was because infection control issues did not always allow CT scans to be taken immediately after the initial respiratory mechanics measurements. Fifth, recruitability was stratified by the median R/I ratio of this analysis population, which may not necessarily be the optimal stratification method. To the best of our knowledge, the R/I ratio cut-off values have been reported based on the median value^8,20^, and the same approach was taken in this study. Future studies are needed to clarify the cut-off value in relation to patients’ outcomes. Sixth, this study evaluated C_rs_ and CT analysis of the global lung. Given the regional heterogeneity of ARDS lung, further studies using imaging techniques, including electrical impedance tomography, are warranted^21,22^.

## Conclusions

In this study, C_rs_ at the applied PEEP was significantly correlated with normally aerated lung volume and tissue on CT. Moreover, recruitability indicated by the R/I ratio and C_rs_ were significantly associated with the normally aerated lung volume. This research underscores the significance of C_rs_ at the applied PEEP as a bedside-measurable parameter and sheds new light on the link between recruitability and normally aerated lung.

### Supplementary Information

Supplementary Figure 1.

Supplementary Figure 2.

Supplementary Figure 3.

Supplementary Figure 4.

Supplementary Tables.

Supplementary Table 3.

Supplementary Legends.

## Data Availability

The datasets used and/or analyzed in the current study are available from the corresponding author upon reasonable request.

## Abbreviations

CT: Computed tomography
ARDS: Acute respiratory distress syndrome
C_rs_: Respiratory system compliance
PV curve: Pressure–volume curve
ZEEP: Zero positive end-expiratory pressure
PEEP: Positive end-expiratory pressure
R/I ratio: Recruitment-to-inflation ratio
ICU: Intensive care unit
AOP: Airway opening pressure
ΔEELV: Change in end-expiratory lung volume between two PEEP levels
ΔPrec: Pressure over which recruitment is assessed
ΔVrec: Recruited volume
Crec: Compliance of the recruited lung
HU: Hounsfield unit scale
BMI: Body mass index
P/F ratio: PaO_2_/F_i_O_2_ ratio
SOFA score: Sequential organ failure assessment score
IQR: Interquartile range
CI: Confidence interval
