# Supplementary figures and images for: Correlation between normally aerated lung and respiratory system compliance at clinical high positive end-expiratory pressure in patients with COVID-19
Source: Sci Rep. 2024 Jun 24;14:14477. doi: 10.1038/s41598-024-64622-3 (PMC11196724; doi:10.1038/s41598-024-64622-3)

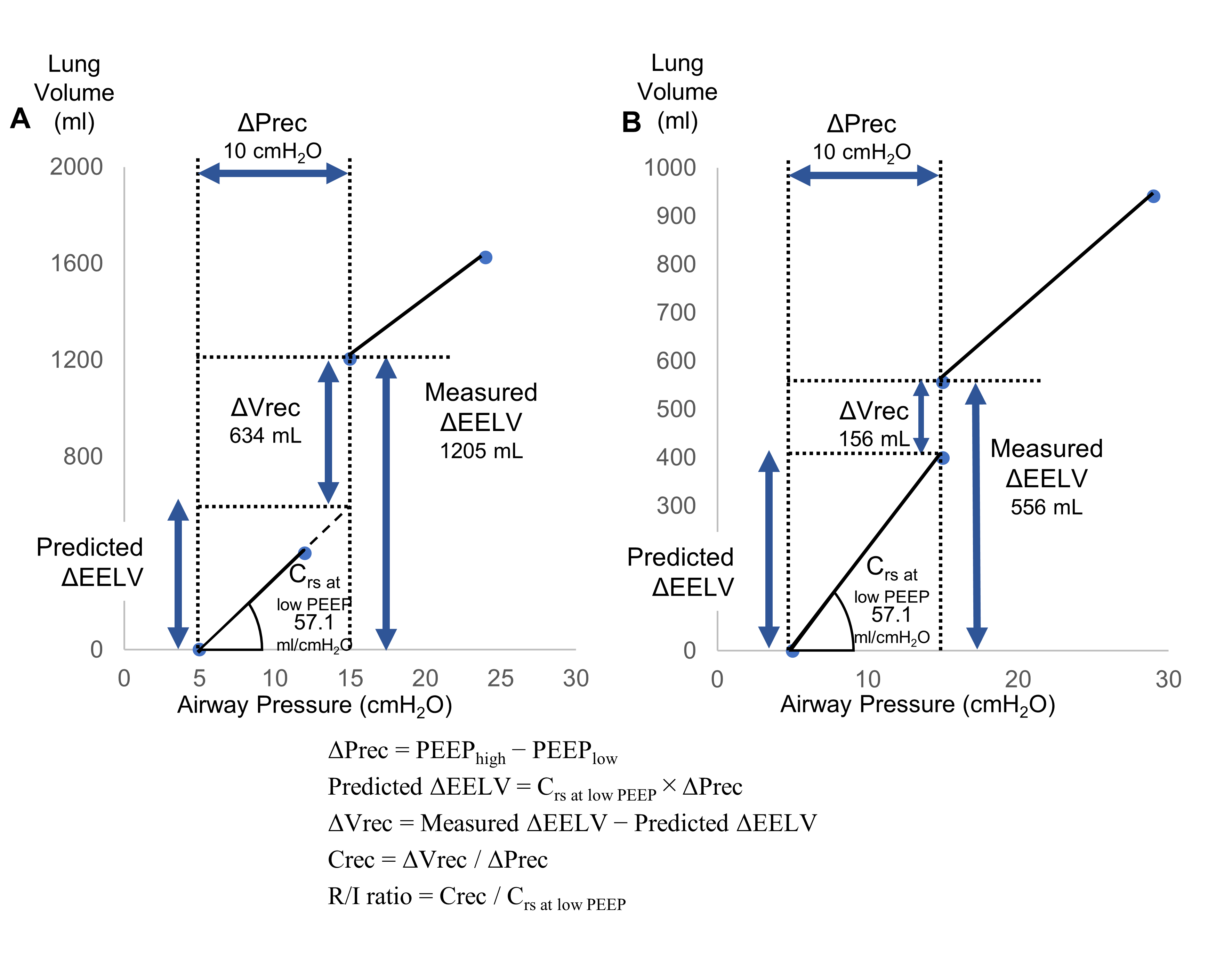

Supplement: Supplementary file 1 — Supplementary Figure 1. [file 41598_2024_64622_MOESM1_ESM.tif]

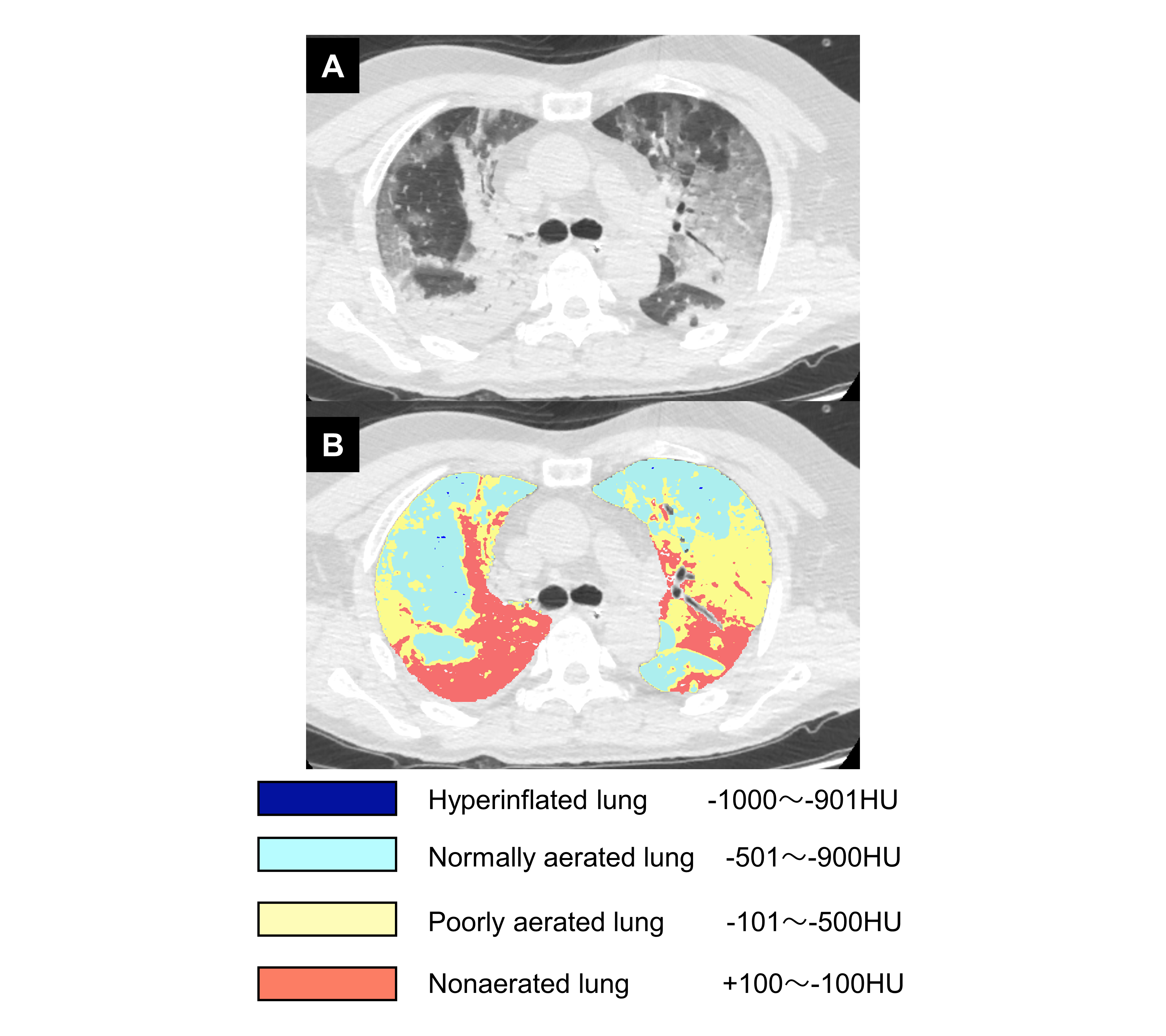

Supplement: Supplementary file 2 — Supplementary Figure 2. [file 41598_2024_64622_MOESM2_ESM.tif]

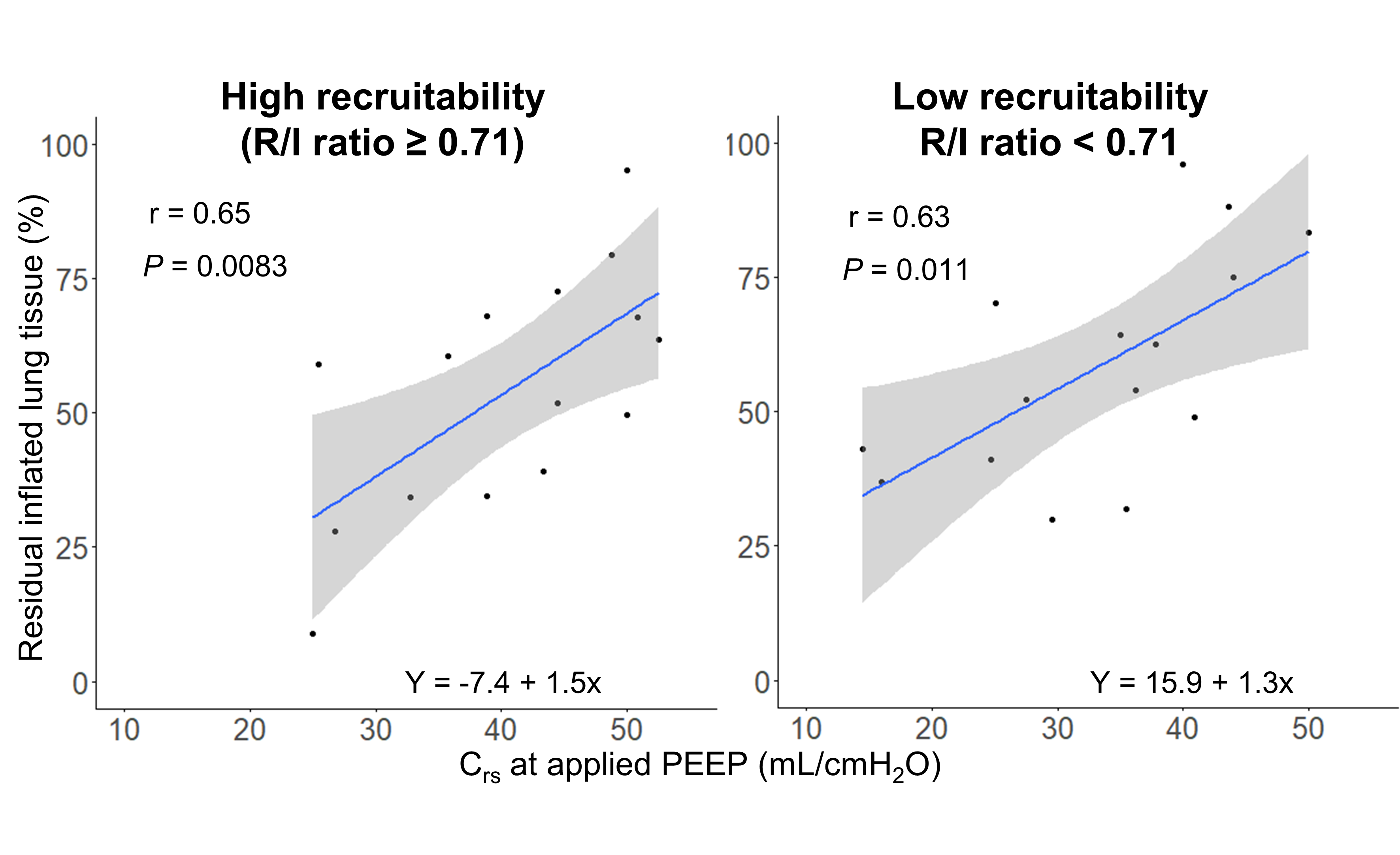

Supplement: Supplementary file 3 — Supplementary Figure 3. [file 41598_2024_64622_MOESM3_ESM.tif]

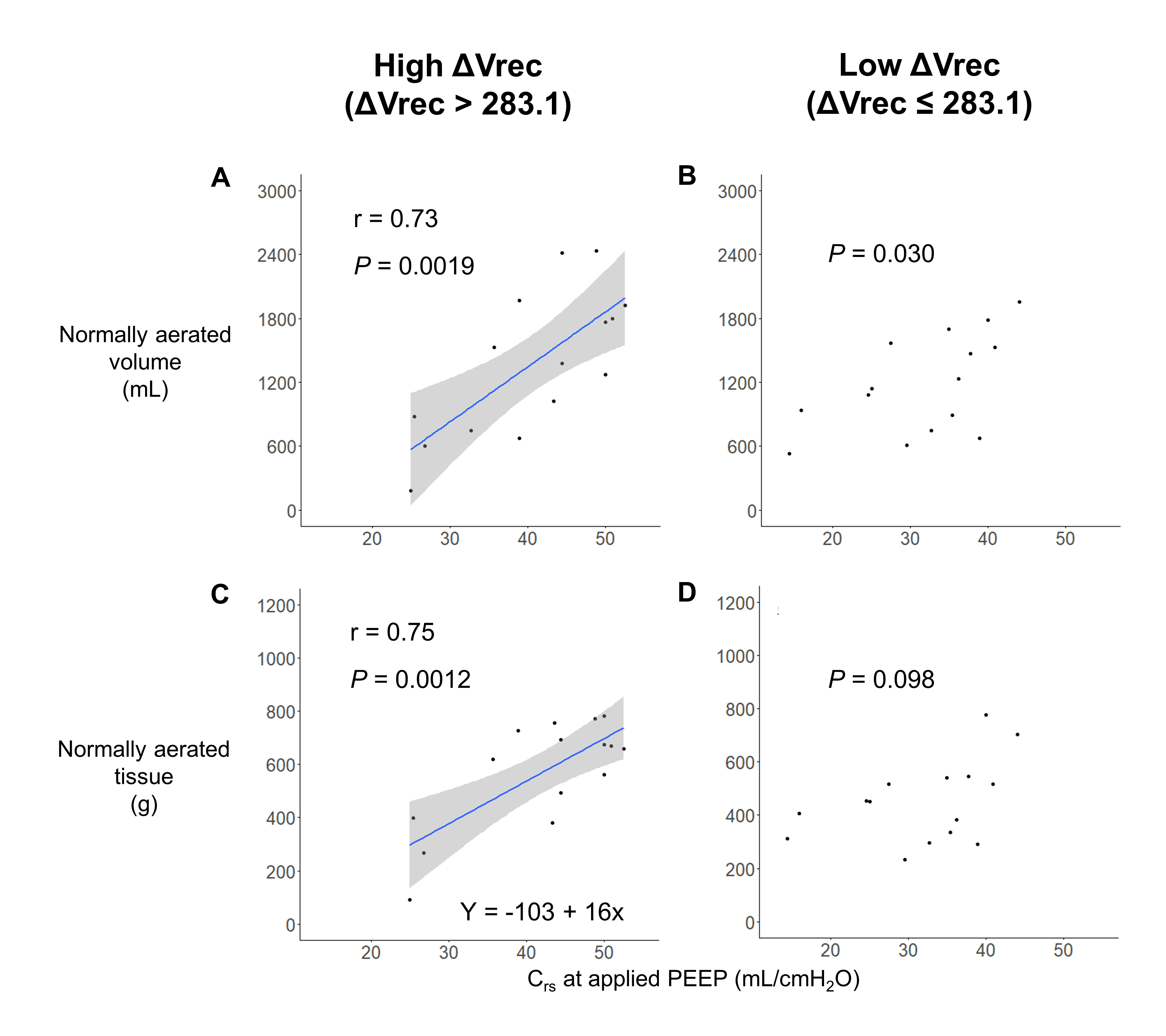

Supplement: Supplementary file 4 — Supplementary Figure 4. [file 41598_2024_64622_MOESM4_ESM.tif]
