# Supplementary material for: Correlation between normally aerated lung and respiratory system compliance at clinical high positive end-expiratory pressure in patients with COVID-19
Source: Sci Rep. 2024 Jun 24;14:14477. doi: 10.1038/s41598-024-64622-3 (PMC11196724; doi:10.1038/s41598-024-64622-3)
Supplement: Supplementary file 5 — Supplementary Tables. [file 41598_2024_64622_MOESM5_ESM.docx]

Supplementary Table 1. Patient characteristics and the division of the groups according to the median recruited volume (ΔVrec).

|  | Total | ΔVrec > 283.1 | ΔVrec ≤ 283.1 |
| --- | --- | --- | --- |
|  | N = 30 | N = 15 | N = 15 |
| Age, years | 63.5 [51.0, 66.0] | 64.0 [50.0, 65.5] | 63.0 [51.5, 68.0] |
| Sex, men | 22 (73.3) | 12 (80.0) | 10 (66.7) |
| Height, cm | 169.0 [162.0, 172.8] | 170.0 [168.5, 172.5] | 163.5 [159.0, 173.0] |
| Body weight, kg | 77.3 [67.0, 89.7] | 84.0 [67.5, 89.4] | 74.0 [68.0, 89.3] |
| Body mass index, kg/m^2^ | 27.9 [23.8, 29.6] | 28.0 [23.9, 29.9] | 27.1 [23.5, 29.4] |
| Predicted body weight, kg | 65.1 [55.3, 68.5] | 66.0 [64.7, 68.3] | 58.7 [53.8, 68.7] |
| KL-6 at admission, U/mL | 464.0 [355.5, 745.5] | 415.0 [359.0, 609.0] | 565.0 [386.0, 953.0] |
| Receiving ECMO, n (%) | 4 (13.3) | 2 (13.3) | 2 (13.3) |
| Receiving prone position, n (%) | 29 (96.7) | 15 (100.0) | 14 (93.3) |
| Receiving high-flow nasal cannula before mechanical ventilation, n (%) | 3 (10.0) | 0 (0.0) | 3 (20.0) |
| Survival at discharge, n (%) | 28 (93.3) | 15 (100.0) | 13 (86.7) |
| Pre-existing conditions, n (%) |  |  |  |
| COPD | 15 (50.0) | 6 (40.0) | 9 (60.0) |
| Hypertension | 12 (40.0) | 7 (46.7) | 5 (33.3) |
| Diabetes | 26 (86.7) | 12 (80.0) | 14 (93.3) |
| SOFA score at R/I ratio measurement | 5.5 [3.2, 7.0] | 6.0 [4.5, 7.0] | 5.0 [2.5, 8.0] |
| PaO_2_/F_i_O_2_ at recruitability assessment, mmHg | 119.7 [102.2, 193.8] | 165.0 [103.5, 227.2] | 109.0 [91.6, 157.1] |
| Respiratory data at R/I ratio measurement |  |  |  |
| TV, mL | 395.0 [350.0, 416.8] | 400.0 [400.0, 484.0] | 350.0 [328.5, 387.5] |
| TV/PBW, mL/kg | 6.2 [5.9, 6.8] | 6.2 [6.1, 7.3] | 6.0 [5.7, 6.4] |
| Respiratory rate, b/min | 16.0 [15.0, 18.0] | 16.0 [14.0, 18.0] | 16.0 [15.0, 18.0] |
| P_plat_, cmH_2_O | 25.0 [23.0, 27.8] | 25.0 [23.0, 27.0] | 25.0 [22.5, 27.5] |
| PEEP, cmH_2_O | 15.0 [12.2, 15.8] | 15.0 [14.5, 16.0] | 13.0 [12.0, 15.0] |
| C_rs_, mL/cmH_2_O | 38.3 [28.0, 44.3] | 44.4 [37.3, 50.0] | 35.0 [26.3, 38.3] |
| AOP > 5 cmH_2_O, n (%) | 4 (13.3) | 2 (13.3) | 2 (13.3) |
| R/I ratio | 0.71 [0.52, 0.88] | 0.88 [0.82, 1.35] | 0.52 [0.42, 0.62] |
| ∆Vrec, mL | 283.1 [191.0, 348.5] | 356.0 [315.1, 493.2] | 189.0 [129.7, 217.2] |
| Measured ∆EELV, mL | 634.0 [518.0, 761.8] | 764.0 [687.5, 934.0] | 510.0 [421.5, 584.5] |
| Crec, mL/cmH_2_O | 28.3 [20.1, 34.9] | 35.6 [31.5, 55.2] | 19.7 [13.3, 22.4] |
| Lung analyses data on CT |  |  |  |
| Interval between onset and CT scans, day | 10.0 [7.2, 12.0] | 11.0 [8.0, 12.0] | 9.0 [7.5, 13.0] |
| Hyperinflated lung tissue, g | 11.3 [5.7, 22.0] | 10.9 [5.9, 23.6] | 11.8 [6.3, 18.5] |
| Normally aerated lung tissue, g | 515.7 [380.1, 673.6] | 658.6 [446.5, 709.8] | 451.8 [324.2, 527.8] |
| Poorly aerated lung tissue, g | 488.0 [342.5, 553.1] | 503.7 [318.7, 559.2] | 472.3 [384.5, 542.1] |
| Nonaerated lung tissue, g | 221.9 [116.1, 375.5] | 184.6 [94.9, 327.6] | 251.7 [163.8, 387.5] |
| Hyperinflated lung volume, mL | 147.8 [70.6, 294.4] | 131.7 [77.8, 297.3] | 159.4 [75.1, 274.2] |
| Normally aerated lung volume, mL | 1422.9 [903.9, 1792.7] | 1761.3 [1148.5, 1995.1] | 1142.7 [820.0, 1546.4] |
| Poorly aerated lung volume, mL | 246.0 [164.5, 267.5] | 251.0 [152.8, 278.6] | 241.1 [193.6, 265.0] |
| Nonaerated lung volume, mL | 6.2 [4.3, 8.7] | 5.4 [3.6, 7.6] | 7.2 [4.5, 9.3] |
| Residual inflated lung tissue, % | 56.5 [39.6, 69.6] | 63.5 [50.6, 76.0] | 49.0 [35.7, 63.4] |

Continuous variables were expressed as median [interquartile range]. Categorical variables were expressed as numbers and proportions.

KL-6, Krebs von den Lungen-6; ECMO, extracorporeal membrane oxygenation; COPD, chronic obstructive pulmonary disease; SOFA, Sequential Organ Failure Assessment; TV, tidal volume; PBW, predicted body weight; Pplat, plateau pressure; PEEP, positive end-expiratory pressure; Crs, respiratory system compliance; AOP, airway opening pressure; R/I ratio, recruitment-to-inflation ratio; ΔVrec, recruited volume; ∆EELV, change in end-expiratory lung volume; Crec, compliance of the recruited lung; CT, computed tomography

Supplementary Table 2. Multivariable linear regression analysis validating the association between respiratory system compliance and normally aerated lung volume and tissue, incorporating recruitability adjusted based on the median recruited volume (∆Vrec) of the study population.

|  | Coefficient [95% CI] | |
| --- | --- | --- |
|  | Normally aerated volume (mL) | Normally aerated tissue (g) |
| Intercept | -176.5  [-838.9, 485.9] | 62.2  [-145.2, 269.6] |
| Recruitability  (∆Vrec > 283.1) | -27.2  [-423.3, 368.9] | -4.3  [-128.4, 119.7] |
| Respiratory system compliance | 42.9^***^  [23.5, 62.2] | 12.2^***^  [6.1, 18.2] |

* *P* <0.025 ** *P* <0.005 *** *P* <0.0005

The estimates of the regression coefficients are showed with 95% confidence interval by multivariate linear regression analysis. Recruitability was stratified into two groups, high and low recruitability, based on the median recruited volume (∆Vrec) of the study population.

CI, confidence interval; ΔVrec, recruited volume
