# Supplementary material for: Correlation between normally aerated lung and respiratory system compliance at clinical high positive end-expiratory pressure in patients with COVID-19
Source: Sci Rep. 2024 Jun 24;14:14477. doi: 10.1038/s41598-024-64622-3 (PMC11196724; doi:10.1038/s41598-024-64622-3)
Supplement: Supplementary file 6 — Supplementary Table 3. [file 41598_2024_64622_MOESM6_ESM.docx]

Supplementary Table 3. Matrix of Pearson's correlation coefficients, including respiratory mechanics and lung analysis items on computed tomography.

|  | P/F ratio | C_rs_ | R/I ratio | Measured  ΔEELV | ΔVrec | Crec | Hyperinflated air volume | Normally aerated air volume | Poorly aerated air volume | Normally aerated tissue | Poorly aerated tissue | Nonaerated tissue | Residual inflated lung tissue |
| --- | --- | --- | --- | --- | --- | --- | --- | --- | --- | --- | --- | --- | --- |
| P/F ratio |  |  |  |  |  |  |  |  |  |  |  |  |  |
| C_rs_ | 0.37^*^ |  |  |  |  |  |  |  |  |  |  |  |  |
| R/I ratio | 0.06 | 0.1 |  |  |  |  |  |  |  |  |  |  |  |
| Measured  ΔEELV | 0.36 | 0.72^***^ | 0.49^**^ |  |  |  |  |  |  |  |  |  |  |
| ΔVrec | 0.26 | 0.54^**^ | 0.79^***^ | 0.91^***^ |  |  |  |  |  |  |  |  |  |
| Crec | 0.27 | 0.49^**^ | 0.82^***^ | 0.87^***^ | 0.98^***^ |  |  |  |  |  |  |  |  |
| Hyperinflated air volume | 0.24 | 0.36 | -0.23 | 0.38^*^ | 0.16 | 0.12 |  |  |  |  |  |  |  |
| Normally aerated air volume | 0.47^***^ | 0.7^***^ | -0.16 | 0.63^***^ | 0.35 | 0.33 | 0.56^**^ |  |  |  |  |  |  |
| Poorly aerated air volume | -0.36 | -0.2 | 0.23 | -0.09 | 0.06 | 0.07 | -0.38^*^ | -0.16 |  |  |  |  |  |
| Normally aerated tissue | 0.39^*^ | 0.67^***^ | -0.09 | 0.62^***^ | 0.39^*^ | 0.39^*^ | 0.46^**^ | 0.95^***^ | 0.06 |  |  |  |  |
| Poorly aerated tissue | -0.4^*^ | -0.33 | 0.25 | -0.18 | 0 | 0.01 | -0.48^**^ | -0.33 | 0.96^***^ | -0.15 |  |  |  |
| Nonaerated tissue | -0.35 | -0.4^*^ | 0.08 | -0.29 | -0.16 | -0.17 | -0.51^**^ | -0.60^***^ | 0.27 | -0.57^**^ | 0.47^**^ |  |  |
| Residual inflated lung tissue | 0.25 | 0.56^**^ | -0.1 | 0.48^**^ | 0.29 | 0.32 | 0.44^*^ | 0.84^***^ | 0 | 0.91^***^ | -0.22 | -0.66^***^ |  |

^*^ *P* <0.05, ^**^ *P* <0.01, ^***^ *P* <0.001

P/F, PaO_2_/F_i_O_2_; C_rs_, respiratory system compliance; R/I ratio, recruitment–to–inflation ratio; ΔEELV, change in end-expiratory lung volume between two PEEP levels; ΔVrec, Recruited volume; Crec, compliance of the recruited lung.
