# Supplementary material for: Correlation between normally aerated lung and respiratory system compliance at clinical high positive end-expiratory pressure in patients with COVID-19
Source: Sci Rep. 2024 Jun 24;14:14477. doi: 10.1038/s41598-024-64622-3 (PMC11196724; doi:10.1038/s41598-024-64622-3)
Supplement: Supplementary file 7 — Supplementary Legends. [file 41598_2024_64622_MOESM7_ESM.docx]

Supplementary Figure 1: Pressure-volume diagram showing the calculation of the recruitment–to–inflation ratio using the single breath method in two cases without airway opening pressure.

The R/I ratio was derived by reducing PEEP from a higher to a lower pressure (from 15 cmH_2_O to 5 cmH_2_O, or 18 cmH_2_O to 8 cmH_2_O) using a single breath method after confirming the presence of an airway opening pressure (AOP) of >5 cmH_2_O. In the case without AOP, for example, the measured change in end-expiratory lung volume between two PEEP levels (measured ΔEELV) is determined from the exhaled breath when the PEEP is dropped from 15 cmH_2_O to 5 cmH_2_O. Predicted ΔEELV is calculated by multiplying the C_rs_ at low PEEP by the pressure over which recruitment is assessed (ΔPrec). Recruited volume (ΔVrec) is calculated by subtracting predicted ΔEELV from measured ΔEELV. Compliance of the recruited lung (Crec) is defined as the ΔVrec divided by the ΔPrec. The R/I ratio is defined as the Crec divided by the C_rs_ at low PEEP.

As an example, Supplementary Figure 1-A shows a patient with a ΔVrec of 634 mL and an R/I ratio of 1.1, and Supplementary Figure 1-B shows a patient with a ΔVrec of 156 mL and an R/I ratio of 0.39.

Supplementary Figure 2: An example of computed tomography (CT) image (a) and lung area analysis (b) using Synapse Vincent version 6.4 (Fujifilm Corporation, Tokyo, Japan).

The voxels in the whole lungs had a CT number (Hounsfield unit scale [HU]) and were classified into four groups according to the CT number: nonaerated (+100HU to -100 HU), poorly aerated (-101 to -500 HU), normally aerated (-501 to -900 HU), and hyper-inflated (-1000 HU to -901 HU).

Supplementary Figure 3:

Correlation of the C_rs_ at the clinical setting of PEEP with residual inflated lung tissue when divided into two groups according to recruitability indicated by the median R/I ratio of 0.71 (High recruitability group: r = 0.65, *P* = 0.0083, Low recruitability group: r = 0.63, *P* = 0.011).

Crs, static respiratory system compliance; PEEP, positive end-expiratory pressure; R/I, recruitment–to–inflation ratio

Supplementary Figure 4: The relationship between the C_rs_ at the clinical setting of PEEP and normally aerated lung volume and tissue when divided into two groups by the median ΔVrec.

In the high ΔVrec group, C_rs_ was correlated with normally aerated lung volume (r = 0.73 [95% CI, 0.35 to 0.91], *P* = 0.0019) and tissue (r = 0.75 [95% CI, 0.39 to 0.91], *P* = 0.0012). In the low ΔVrec group, C_rs_ was not correlated with normally aerated lung volume or tissue.

C_rs_, static respiratory system compliance; PEEP, positive end-expiratory pressure; CI, confidence interval
